# Health-related quality of life in girls and boys with juvenile idiopathic arthritis: self- and parental reports in a cross-sectional study

**DOI:** 10.1186/1546-0096-10-33

**Published:** 2012-09-17

**Authors:** Veronica Lundberg, Viveca Lindh, Catharina Eriksson, Solveig Petersen, Eva Eurenius

**Affiliations:** 1 Department of Clinical Sciences, Pediatrics, Umeå University, Umeå, Sweden; 2 Department of Nursing, Umeå University, Umeå, Sweden; 3 Department of Clinical Sciences, Child and Adolescent Psychiatry, Umeå University, Umeå, Sweden; 4 Department of Public health and Clinical medicine, Epidemiology and Global health, Umeå University, Umeå, Sweden

**Keywords:** Adolescent, Child, Gender, Parents, Pediatrics, Questionnaire

## Abstract

**Background:**

Juvenile Idiopathic Arthritis (JIA) affects children and adolescents with both short-term and long-term disability. These children also report lower health-related quality of life (HRQOL) compared to their healthy peers. However, there seems to be some discrepancies between self- and parent-reports, and gender differences need to be further studied. This study aims to describe HRQOL in girls and boys with JIA, and to explore gender differences in self-reports compared to parent-reports of HRQOL in children with JIA.

**Methods:**

Fifty-three children and adolescents with JIA (70% girls and 30% boys) with a median age of 14 years (8–18 years), and their parents, participated in this cross-sectional study in Sweden. Data was systematically collected prior to ordinary visits at a Pediatric outpatient clinic, during a period of 16 months (2009–2010). Disability was assessed with the Childhood Health Assessment Questionnaire (CHAQ), and disease activity by physicians’ assessments and Erythrocyte Sedimentation Rate (ESR). The Pediatric Quality of Life Inventory 4.0 Generic Core Scales (PedsQL) was used to assess self- and parent-reports of HRQOL in the child.

**Results:**

In this sample of children with generally low disease activity and mild to moderate disability, more than half of the children experienced suboptimal HRQOL, equally in girls and boys. Significant differences between self- and parent-reports of child HRQOL were most evident among girls, with lower parent-reports regarding the girl’s physical- and psychosocial health as well as in the total HRQOL score. Except for the social functioning subscale, where parents’ reports were higher compared to their sons, there were no significant differences between boys- and parent-reports.

**Conclusions:**

More than half of the girls and boys experienced suboptimal HRQOL in this sample, with no gender differences. However, there were differences between self- and parent-reports of child HRQOL, with most significant differences found among the girls. Thus, differences between self- and parent-reports of child HRQOL must be taken into account in clinical settings, especially among girls with JIA.

## Background

The yearly incidence of Juvenile Idiopathic Arthritis (JIA) in Sweden is about 15 per 100 000 children, and JIA is more common among girls than boys [1]. Although JIA is a collective term for chronic rheumatic diseases in children, it is frequently used as one diagnosis. JIA is an important cause of short-term and long-term disability [2]. The disease affects physical functioning and mental health, and also impact school and home functioning [3]. As children reach adulthood, they face possible continuing disease activity, medication-associated morbidity, life-long disability, and an increased risk of emotional and social dysfunctioning [3].

It has also been established that children with JIA report lower health-related quality of life (HRQOL) as compared to their healthy peers [3-9]. Quality of life (QOL) is generally conceptualized as a broad concept, addressing well-being across various domains. HRQOL is considered a sub domain of the more global QOL construct. However, there are a range of definitions, theories, domains, and items associated with pediatric QOL assessment [10]. In the present study, the following HRQOL definition was utilised: ‘a multidimensional construct covering physical, mental, social, and behavioural components of well-being and function as perceived by the patients, and/or individual feelings associated with health’ [10]. The consequences of JIA seem to influence HRQOL, with physical functioning being most affected [3,9,11]. Disability and pain have been found to be the most important determinants of physical and psychosocial well-being, in a European and Latin American study of HRQOL in JIA patients [9].

Instruments used to measure HRQOL can be condition-specific, generic, or chronic generic. The condition-specific instruments address aspects related to a specific condition and can be used to compare data from children and adolescents with the same chronic medical condition. Generic instruments are usable for assessment in all children, whether they are healthy or suffer from a medical condition [12]. Generic instruments for chronic disease are usable in children and adolescents who suffer from any chronic medical condition, and permit comparisons across different medical conditions while taking into account specific areas affected by a chronic disease [13]. Pediatric HRQOL-instruments must be sensitive to cognitive development and can include both child self-reports and parent proxy-reports [14]. However, it is important to obtain self-reports whenever possible, as there seem to be some differences between self- and parent-reports of HRQOL in children [14-16].

It is not sufficient to know if a medical treatment can save lives or hold diseases at bay. How the treatment affects patient’s HRQOL is also important in medical evaluations [17]. Health care professionals need insight into the HRQOL of patients with JIA, to better understand their overall health situation, and thus, have a better basis for performing targeted interventions. HRQOL in children and adolescents with JIA in northern Sweden has not earlier been systematically studied. Studies examining gender discrepancies between self- and parent-reports in JIA also seem to be lacking. Our assumption was that girls would report lower HRQOL than boys, as earlier findings from a community-based study indicated that, from early adolescence females develop internalizing symptoms (e.g. depression) at higher rates than males [18]. We also assumed that both girls and boys equally would report higher HRQOL in comparison to parent-reports of their HRQOL, as found in other studies [15,19]. In the light of the discrepancies between self- and parent-reports, also gender differences need to be further studied.

### Aim

The aim of this study was to describe HRQOL in girls and boys with JIA in Sweden, and to explore gender differences in self-reports compared to parent-reports of HRQOL in children with JIA.

## Methods

### Study design and study population

A cross-sectional questionnaire survey approaching children and adolescents registered with a JIA diagnosis at the Department of Child and Adolescents Medicine, Norrland’s University Hospital in Umeå, Sweden. The department serviced all children with JIA in the county of Västerbotten and the study population included all 8–18 year olds (n = 69) with routine outpatient appointments between September 2009 and December 2010.

### Participants

Out of the 69 eligible children identified through clinical appointment schedules, 56 children and their parents agreed to participate in the study. Three were excluded due to missing parent-reports and therefore 53 children (37 girls and 16 boys) remained to be studied. Of the participating parents, 37 were mothers, 12 fathers, and in four families, the mothers and fathers jointly completed the questionnaires. The reason for declining participation was unknown in six families, four parents expressed that the child only had minor problems due to the disease, two failed to participate due to a lack of time, and one child was transitioned into adult care. Eligibility criteria was child age 8 to 18 years and a JIA diagnosis as defined by the International League of Associations for Rheumatology (ILAR) classification criteria [20].

### Measures

*HRQOL* was assessed using the Pediatric Quality of Life Inventory 4.0 Generic Core Scales (PedsQL) [14]. This generic questionnaire consisted of 23 items combined into physical, emotional, social, and school functioning scales. A physical health summary score was based on eight items and equal to the physical functioning scale. A psychosocial health summary score was based on the 15 items from the emotional, social and school functioning scales. Mean values were computed for each scale. In this study the self- and parent-report forms for ages 8 to 12 years, and 13 to 18 years were used. The parent-report forms assessed the parent’s perception of HRQOL in their children and were constructed to directly parallel the self-report forms. Recall time was one month and a five point response scale was utilized with alternatives between 0 (never a problem) and 4 (almost always a problem). Items scores were reversed and linearly transformed to a 0 to 100 scale (0 = 100, 1 = 75, 2 = 50, 3 = 25, 4 = 0), with higher PedsQL scores indicating better HRQOL [14]. Suboptimal HRQOL was defined as a PedsQL total mean scale score <78.6 [8]. Reliability and validity has been tested in diverse samples of healthy children and pediatric patients with acute or chronic conditions, and PedsQL has been proven reliable, valid and sensitive to disease severity, and responsive to changes in JIA [14]. The Swedish version of PedsQL has also been proved to have acceptable reliability and validity [21]. However, construct validity was questioned for the Swedish proxy form, but not for the self-report form [21].

*Disability* was measured by the Childhood Health Assessment Questionnaire (CHAQ) [22]. The CHAQ comprised 30 items and measured eight functional areas: dressing and grooming, arising, eating, walking, hygiene, reach, grip, and activities. Three components were assessed in each area: a) the degree to which daily functions were difficult to perform, b) the use of special aids or devices, and c) activities for which assistance of another person was required. Each question was scored on a four point scale between 0 (no difficulty) and 3 (unable to do). The question with the highest score determined the score for that functional area. If aids or devices were used or help was needed to complete tasks in a certain area, a minimum score of two points were recorded for the corresponding functional area. The scores for each of the eight functional areas were averaged to calculate a Disability Index (DI), ranging from 0 (no disability) to 3 (disabled) [22]. The cut-off levels for mild, mild-to-moderate, and moderate disability have been suggested at medians 0.13, 0.63, and 1.75, respectively [23]. The CHAQ also assessed pain and overall well-being measured by two 0–100 mm Visual Analogue Scales (pain VAS and well-being VAS), number of days absent from school the last two months due to JIA, and attendance at physical education classes, categorized into three groups; 1 = always, 2 = sometimes, and 3 = not at all [22]. The original CHAQ as well as the Swedish version have been shown to be reliable and valid tools for functional, physical, and psychosocial assessments in children with JIA [22,24,25].

*Disease duration* in years was estimated based on data registered in the medical records. If date of diagnosis was missing, the 1^st^ of the month was registered. If month was missing the 1^st^ of January the year of diagnosis was registered.

*Disease activity* was measured by Erythrocyte Sedimentation Rate (ESR) in mm/hour and by the responsible physician’s assessment of disease activity categorized into; no-, mild-, moderate-, or high disease activity. Moderate disease activity indicated need for changes of medical therapy, mild was defined between no activity and moderate activity, and high activity indicated severe immunologic and clinical activity.

*Medications* were registered in four categories: 1) Disease modifying anti-rheumatic drug (DMARD) e.g. cyclosporine, azathioprin, methotrexate, hydroxychoroquine, and sulphasalazine, 2) Non-steroid anti-inflammatory drug (NSAID) e.g. diclophenac, ibuprofen, naproxen, ketoprofen, 3) Biological medication e.g. etanercept, adalimumab, infliximab, anakinra and rituximab, and 4) Corticosteroid therapy (Table 1).

**Table 1:** Characteristics of 53 children with JIA aged 8–18 years

	**Girls n = 37**	**Boys n = 16**	**p-value**^1^
**Age at study visit**, years Md (range)	14 (8–18)	14 (10–18)	0.612
**CHAQ Disability Index**^2^, n (%)			
No-mild	12 (32)	5 (31)	0.841
Mild- moderate	9 (24)	5 (31)	
Moderate	12 (32)	6 (37)	
Severe	3 (8)		
Missing	1 (3)		
**Pain VAS**^3^, 0–100 Md (range)	31 (0–93)	51 (0–99)	0.377
**Well-being VAS**^4^, 0–100 Md (range)	21 (0–99)	28 (0–100)	0.488
**School absence days**^5^, Md (range)	0 (0–14)	0 (0–6)	0.258
**Physical education attendance,** n (%)			
Always	12 (32)	9 (56)	0.105
Sometimes	15 (41)	5 (31)	
Not at all	6 (16)	2 (12)	
Not relevant	3 (8)		
Missing	1 (3)		
**Disease duration,** years Md (range)	5 (0–16)	2 (0–13)	0.054
**ESR**^6^, mm/hour Md (range)	5 (2–31)	4 (2–35)	0.175
**Disease activity categories**, n (%)			
No	12 (32)	9 (56)	0.221
Low	20 (54)	5 (31)	
Moderate	4 (11)	2 (12)	
High	0	0	
Missing	1 (3)		
**Use of medications**^7^**,** n (%)			
DMARD	24 (65%)	7 (44%)	0.704
NSAID	25 (68%)	8 (50%)	0.416
Biologic medication	4 (11%)	0	
Corticosteroid therapy	7 (19%)	0	

^1^Mann Whitney U test.

^2^Childhood Health Assessment Questionnaire Disability Index.

^3^Visual Analogue Scale for pain during the past week; 0 = no pain and 100 = very severe pain.

^4^Visual Analogue Scale for assessment of overall well-being; 0 = very good and 100 = very bad.

^5^Number of days absent from school the last two months, due to rheumatic disease.

^6^Erythrocyte Sedimentation Rate.

^7^Disease modifying anti-rheumatic drug **(**DMARD), Non-steroid anti-inflammatory drug (NSAID). Participants might have more than one medication.

### Procedure

Families who gave consent to participate in the study completed several questionnaires as parts of a larger study, of which the current study report HRQOL results based on the PedsQL questionnaire. The questionnaires were answered at the clinic prior to a scheduled visit to a Pediatric rheumatologist. Parents and children completed the PedsQL separately, with a research assistant available for the child. The CHAQ was used routinely in clinical practice, and was answered by children and their parents at home before visiting the Pediatric outpatient clinic. The PedsQL has been reported to take approximately five minutes to complete [14] and the CHAQ less than ten minutes [22]. Estimated time to complete all the study-questionnaires was 20–40 minutes for each child and another ten minutes for the CHAQ to be completed at home before the visit. All questionnaires were numbered with a study-ID, and after completion placed in a locked box, available only for the researcher who was responsible for the study. Disease activity was assessed by the Pediatric rheumatologist.

### Ethical considerations

Informed consent was given by each participant. Reporting was done on group level without revealing results on an individual level. Ethical approval was obtained from the regional ethical review board in Umeå, Sweden (Dnr 09-070 M).

### Statistics

Data from girls and boys were analyzed separately. Results of the descriptive analysis were presented as median and range or numbers (n) and percentages (%). Results regarding HRQOL were presented by mean, standard deviation (SD), median (Md), and range. Mann Whitney U test was used to analyse gender differences, and Wilcoxon signed-rank test for analysis of differences between self- and parent-reports. The analysis was performed by use of the Statistical Package for the Social Sciences (SPSS) version 18 and 19. Statistical significance was defined at p **<** 0.05.

## Results

### Sample characteristics

Median age for the participating children was 14 years (range 8–18 years) and their disease activity was generally low. Girls and boys did not differ significantly in any of the assessed characteristics (Table 1). The following diagnoses were identified among the participants; oligoarthritis, n = 19 (36%), polyarticular arthritis RF-positive, n = 9 (17%), enthesetis-related arthritis, n = 8 (15%), unspecified arthritis, n = 7 (13%), psoriatic arthritis, n = 6 (11%), polyarticular arthritis RF-negative, n = 2 (4%), and systemic arthritis, n = 2 (4%). In ten of the girls, at least one additional diagnosis was reported by the child or the parent, i.e. uveitis, overweight, asthma, dermatitis, Turners syndrome, celiac disease, Crohn’s disease, and attention-deficit/hyperactivity disorder. Likewise, among six of the boys at least one additional diagnosis was reported, i.e. thyroid gland disease, asthma, allergy, panic disorder, Raynaud’s syndrome and lactose intolerance.

### Health-related quality of life

This sample of children with JIA reported a physical health mean score of 68.31 (SD 20.72), a psychosocial health mean score of 78.66 (SD 16.88), and a total HRQOL mean score of 75.06 (SD 16.28). The parental mean score of the children’s physical health was 66.01 (SD 20.49), the psychosocial health mean score was 73.83 (SD 19.54), and the PedsQL total mean score was 71.14 (SD 18.66). Suboptimal HRQOL was reported by 29 of the children (55%) and 31 of the parents (59%). No gender differences were found in the children’s self-reports. Significant differences between self- and parent-report were most evident among girls, with lower parent-reports compared to self-reports regarding physical- and psychosocial health as well as in the total HRQOL score. There were no significant differences between self- and parent ratings in boys, except for parent-reports of their son’s social functioning being higher than the corresponding self-reports (Table 2). In this subscale, the parents of the boys also reported significantly higher scores compared to the girls’ parents (p = 0.04), but this was not found in any of the other subscales.

**Table 2:** Health-related quality of life measured by PedsQL 4.0 Generic Core Scales in children with JIA aged 8–18 years and differences in self- and parent-reports

	**Girls n = 37 Mean (SD) Median (range)**	**Girls’ parents n = 37 Mean (SD) Median (range)**	**p-value**^**1**^	**Boys n = 16 Mean (SD) Median (range)**	**Boys’ parents n = 16 Mean (SD) Median (range)**	**p-value**^**1**^
Physical health	70.82 (17.98) 68.75 (26.79-100)	65.49 (20.73) 67.86 (31.25-100)	0.028	62.50 (25.72) 65.62 (15.63-100)	67.21 (20.52) 65.62 (18.75-100)	0.116
Emotional functioning	79.86 (21.06) 85.00 (30.00-100)	71.59 (22.78) 75.00 (15.00-100)	0.009	76.48 (23.78) 85.00 (10.00-100)	74.37 (25.02) 77.50 (0.00-100)	0.623
Social functioning	86.69 (14.51) 90.00 (45.00-100)	76.76 (18.49) 80.00 (25.00-100)	0.001	79.37 (19.91) 80.00 (15.00-100)	85.94 (19.25) 90.00 (25.00-100)	0.027
School functioning	71.65 (21.03) 80.00 (25.00-95.00)	67.89 (22.60) 75.00 (20.00-100)	0.135	75.00 (18.07) 80.00 (25.00-100)	73.44 (23.57) 77.50 (5.00-100)	0.637
Psychosocial health	79.40 (15.91) 81.67 (48.33-98.33)	72.06 (18.90) 71.67 (30.00-100)	0.002	76.94 (19.37) 78.75 (16.67-100)	77.92 (20.99) 79.17 (10.00-100)	0.691
**HRQOL total score**	76.44 (14.64) 77.17 (42.61-98.91)	69.81 (18.36) 68.48 (30.43-100)	0.003	71.89 (19.71) 69.02 (16.30-100)	74.20 (19.59) 73.37 (13.04-100)	0.410

^1^Wilcoxon signed-rank test.

## Discussion

Our main finding was that more than half of the children experienced suboptimal HRQOL, based on both self- and parent-reports. No gender differences were found in self-reports of any PedsQL subscale, nor in the total HRQOL scores. On the other hand, there were significant differences between self- and parent-reports, primarely evident among girls.

Children with no, or very mild, clinical JIA-symptoms have been found to experience suboptimal HRQOL, defined as PedsQL total scale score <78.6 [8]. On the other hand, another study showed that JIA patients with inactive polyarticular disease reported HRQOL similar to that of healthy controls [7]. However, our results showing that children and parents rated physical functioning the worst, and social functioning the best, was comparable to results found in an earlier psychometric study [26].

In this study we used PedsQL 4.0, a generic questionnaire which enables comparisons across pediatric patients and healthy populations. A disease-specific and/or chronic-generic questionnaire might have given more detailed information about other aspects of health problems caused by a chronic disease like JIA.

Regarding parent–child agreement, a review study concluded that parents of healthy children tended to report higher scores of HRQOL than the children themselves. On the other hand, parents of children with different health conditions tended to underestimate the child’s HRQOL [19]. This is in accordance with our findings among girls, i.e. lower parent-reports of HRQOL compared to corresponding self-reports. This was also found in another study among children with JIA [15]. However, in our study the parents of the boys tended to report higher scores than their sons with regard to physical health, social functioning, psychosocial health and total HRQOL. These differences were not statistically significant except in the social functioning subscale. In contrast, in another study of children with JIA, the authors found no significant differences between the parents and their children’s evaluation of the child’s quality of life [27].

There are varying findings in the literature on health-ratings in children with JIA. In one sample of adolescent with JIA, a wide variation was found in parent–child agreement for different health-related variables including pain, general health, functional ability, and HRQOL. Parent–child agreement was evidently better for all these variables when they were estimated to be mild or severe and variations were most pronounced for more midrange outcomes [16]. This was supported by another study showing a trend toward better agreement between self- and parent-reports among children with an active disease than among those with an inactive disease [7]. It could have been the case in our study sample with general low disease activity. However, there were no gender differences in disability or disease activity in our study, and therefore this could not explain the differences in girls and boys child–parent- reports in our sample. Previous findings show that physical health is particularly important in rheumatic diseases, and the parent–child agreement on this subscale seems to be high [19,28]. In our study significant differences were found in physical health between child- and parent reports among the girls, but not the boys.

No other studies of gender differences in HRQOL-reports among children with JIA and their parents have been performed, as far as we know. Our results might be one additional explanation for some of the divergent findings in child-parents reports, since the results seem to point out some gender differences. The results indicating parent–child differences to be more evident among girls compared to the boys, was somewhat surprising to us, as we did not expect any gender differences in that regard. Instead, we expected longer disease duration to increase parents’ and children’s awareness and consensus of impact of the disease on life situation. However, the girls seemed to have close to significant longer disease duration compared to the boys. A number of other variables not included in this study may have affected the parent–child reports, e.g. mental health and comorbidity. Mental health among our participants may have impacted the results, as adolescents with JIA, who report depressive symptoms, seem to be more likely to disagree with their parents in ratings of disability, pain, and well-being [29]. In this sample there were a remarkable number of self- and parent-reported additional diagnoses among both sexes (10 out of 37 girls and 6 out of 17 boys). As both girls (27%) and boys (35%) had additional diagnosis, it is uncertain whether comorbidity may have influenced the differences between parent and child report. However, the high degree of comorbidity in this sample might affect the study results generalizability for the whole JIA population. Disability measured by the CHAQ was found to be mild to moderate in our sample, which is in accordance with findings in other studies of children with JIA [9,11,15,16,30].

A satisfying number of children (80%) agreed to participate in this study. Oligoarthritis was the most common JIA sub-diagnosis in our sample, which is in accordance with a Nordic JIA incidence study [31]. The fact that four parents expressed that the child did not have much problems due to the disease and therefore declined to participate might have biased our results slightly towards lower ratings of HRQOL. In terms of generalizability, our sample was relatively small and predominantly female, which is similar to the epidemiology of JIA [32]. This fact makes it more difficult to draw reliable conclusions, but not as much among the girls as among the boys. Because of the small sample size, it was not appropriate to analyse sub-groups for both age and gender, which would have given additional age-specific information about the study population.

The differences between child- and parent-reporting emphasize the importance of health care professionals obtaining information about children’s health situation from both parents and children [15]. Although self-report is considered the gold standard for HRQOL assessment, the parents’ perception of their child’s HRQOL influences health care utilization. It is therefore essential to increase the knowledge about the relationship between parent- and child-reporting [17]. Relying solely on parent-reports will only partly inform the health professionals [16]. However, children with JIA have reported difficulties in communicating their problems to health professionals [15]. It is therefore of great importance to support children’s ability to self-report verbally and in writing within pediatric care. PedsQL was simple to complete for the child at the outpatient clinic, but to make the instrument feasible for clinical use; a faster method for generating scale scores is required. Further research is needed to better understand gender differences in child–parent reports of HRQOL in children with JIA, for example in larger samples and through in-depth interviews.

## Conclusions

More than half of the girls and boys experienced suboptimal HRQOL in this sample, with no gender differences. However, significant differences between self- and parent-reports of child HRQOL were shown, primarily among the girls. Thus, differences between child- and parent-reports of HRQOL must be taken into account in clinical settings, especially among girls with JIA.

## Competing interests

All authors have declared that they have no competing interest.

## Authors’ information

VLU, RPT, Centre for Children and Adolescents, University Hospital of Umeå, Sweden.

VLI, PhD, Department of Nursing, Umeå University, Umeå, Sweden.

CE, MD, Centre for Children and Adolescents, University Hospital of Umeå and Department of Clinical Microbiology, Umeå University.

SP, PhD. Department of Clinical Sciences, Child and Adolescent Psychiatry, Umeå University, Umeå, Sweden.

EE, PhD and Health Promotion Officer, Västerbotten County Council and Researcher within the field of Public Health at the Department of Epidemiology and Global Health, Umeå University, Sweden.

## Authors’ contributions

VLU is the corresponding author and has substantially contributed to the study conception and design, in analysis and interpretation of data and in drafting of the manuscript. VLI has substantially participated in the study conception and design, in the analysis and interpretation of data and in editing of the manuscript. CE has substantially contributed in the study conception and design, in the analysis and interpretation of data and in editing of the manuscript. SP has substantially contributed in the study conception and design, in the analysis and interpretation of data, and in editing of the manuscript. EE has substantially contributed in analysis and interpretation of data as well as in drafting and editing of the manuscript. All authors read and approved the final manuscript.
