# Correction to: Health-related quality of life in girls and boys with juvenile idiopathic arthritis: self- and parental reports in a cross-sectional study

**DOI:** 10.1186/s12969-018-0269-2

**Published:** 2018-08-22

**Authors:** Veronica Lundberg, Viveca Lindh, Catharina Eriksson, Solveig Petersen, Eva Eurenius

**Affiliations:** 1 Department of Clinical Sciences, Pediatrics, Umeå University, Umeå, Sweden; 2 Department of Nursing, Umeå University, Umeå, Sweden; 3 Department of Clinical Sciences, Child and Adolescent Psychiatry, Umeå University, Umeå, Sweden; 4 Department of Public health and Clinical medicine, Epidemiology and Global health, Umeå University, Umeå, Sweden

## Correction

Following publication of the original article [1], the authors reported an error in the data of their article: one girl was by mistake scored as a boy. The authors have made new analyses of the corrected data. The corrected data and the new analyses are listed in this Correction.

- The characteristics in table 1 do not differ when it comes to significant values, with the new corrected analyses. None of the parameters, presented in the Table 1, differ significantly. This is the same result as presented in the original article [1].
- In Table 2 the new corrected analyses show that the significant differences between child and parent report still differ significantly, however with other values. The non-significant results still is non-significant with our new analyses.
- However, in the last section in the presentation of Results/ Health-related quality of life we write (regarding social functioning): “In this subscale, the parents of the boys also reported significantly higher scores compared to the girls’ parents (*p*=0.04), but this was not found in any of the other subscales”. This statement is not correct. The *p*-value for differences between the boys’ and girls’ parents report were non-significant (*p*=0.062).

The tables which are cited in the above sentences can be reviewed in the original article [1].

## References

[1] Lundberg V (2012). Health-related quality of life in girls and boys with juvenile idiopathic arthritis: self- and parental reports in a cross-sectional study. Pediatric Rheumatology.

